# Optimizing vitamin A supplementation: A comparative cost-effectiveness analysis of routine distribution strategies in northern Côte d’Ivoire

**DOI:** 10.1371/journal.pone.0338784

**Published:** 2025-12-15

**Authors:** Melissa M. Baker, Lyonel Nerolin Doffou Assalé, David Doledec, Romance Dissieka, Ahmenan Claude Liliane Konan, Agnes Helen Epse Assagou Mobio, Koffi Landry Kouadio, Oka René Kouamé, Ama Emilienne Yao, Hubert Zirimwabagabo

**Affiliations:** 1 Helen Keller Intl, Nairobi, Kenya; 2 Helen Keller Intl, Abidjan, Côte d’Ivoire; 3 Ministère de la santé et de l’hygiène Publique, Abidjan, Côte d’Ivoire; University of Waterloo, CANADA

## Abstract

**Background:**

While recent data on vitamin A deficiency (VAD) prevalence is lacking, the 2004 Côte d’Ivoire Nutrition and Mortality Survey reported that 26.7% of children aged 6–59 months were affected by VAD, and approximately 60% were at risk. Since 2016, the government has transitioned from mass campaigns to routine vitamin A supplementation (VAS) delivery integrated into health services. However, evidence on the cost-effectiveness of the routine distribution approaches is limited. This study evaluated the cost, coverage, and cost-effectiveness of three routine VAS delivery strategies across two health districts in northern Côte d’Ivoire.

**Methods:**

A mixed-methods study evaluated three routine VAS delivery strategies – routine-fixed, advanced community-based, and catch-up – across two health districts, Ferkessédougou and Niakaramadougou, in northern Côte d’Ivoire. The quantitative cost data were collected via a structured tool covering six cost categories: planning, procurement, training, social mobilization, distribution, and supervision. VAS coverage was assessed through a post-event coverage survey (PECS) via a two-stage cluster sampling methodology. A cost-effectiveness analysis determined the cost per child supplemented, the cost per DALY averted, and a sensitivity analysis tested the robustness of the findings under different cost scenarios.

**Results:**

The total program cost for July-December 2023 was 25.5 million FCFA, with personnel costs comprising over 70% of expenditures. In Ferkessédougou, the routine advanced community-based strategy was the most cost-effective, at 458 FCFA per child in rural areas (versus 596 FCFA for the routine-fixed facility-based approach in the same area). In Niakaramadougou, the December catch-up was more cost-effective in rural areas (606 FCFA per child) than the routine-fixed approach (714 FCFA). Across both districts combined, the routine-fixed strategy averaged roughly 651 FCFA per child supplemented, and the cost per DALY averted ranged from 30,093 FCFA (advanced strategy in Ferkessédougou) to 89,550 FCFA (catch-up Jul 2023 in Niakaramadougou) – all below Côte d’Ivoire’s cost-effectiveness threshold (0.5 x GDP per capita; approximately USD 1,265).

**Conclusion:**

All three strategies were cost-effective, though the advanced community-based strategy achieved the best balance of reach and efficiency. Scaling advanced strategies within health system constraints may enhance sustainability and coverage in low-resource settings.

## Background

Micronutrient malnutrition remains a significant public health concern in Côte d’Ivoire, disproportionately affecting young children and hindering their growth and development. In Côte d’Ivoire, 23% of children under five years old are stunted, 8% are wasted, and 14% are underweight [1]. Recent evidence from Uganda demonstrates a strong epidemiologic link between vitamin A deficiency (VAD) and growth faltering: children with VAD had a significantly higher odds of stunting compared to non-deficient peers, while no significant association was found with wasting or being underweight [2]. While recent data on VAD prevalence is lacking, the 2004 Côte d’Ivoire Nutrition and Mortality Survey reported that 26.7% of children aged 6–59 months were affected by VAD, and approximately 60% were at risk [3].

In Côte d’Ivoire, vitamin A supplementation (VAS) has been central to efforts to combat VAD, recognized for its potential to reduce all-cause mortality by up to 24% in children aged 6–59 months [4]. Historically, mass immunization campaigns were the primary mechanism for VAS delivery, achieving high coverage rates, particularly in rural and underserved regions with limited health service access. These campaigns incorporated door-to-door visits by community health workers (CHWs) to administer doses and substantial community mobilization efforts to ensure equitable distribution. Coverage rates during these campaigns frequently exceeded 90% in some districts [5].

Despite their success, mass campaigns are costly and rely heavily on external funding, thus raising concerns about their long-term sustainability [6]. External factors such as climate change, global health crises, and political instability have also disrupted campaigns, further straining health systems [7,8]. To address these challenges, the government of Côte d’Ivoire began transitioning to routine VAS delivery in 2016, integrating supplementation into the primary health system.

By December 2024, 73 out of the 113 health districts in Côte d’Ivoire had adopted a mixed model of routine VAS delivery, utilizing fixed, advanced, and catch-up strategies. Routine-fixed strategies target children attending health facilities for vaccination or growth monitoring services. Advanced strategies involve community-based distribution at accessible locations within 5–15 kilometers of health centers. The catch-up delivery strategy, consisting of a 4-day door-to-door distribution event, aims to distribute to children who live in areas with low coverage at the end of the semester [5].

Given these evolving strategies, evaluating their effectiveness in delivering VAS is crucial. This study aimed to measure the cost, coverage, and cost-effectiveness of the three routine VAS distribution approaches.

## Materials and methods

### Study design

This study employed a mixed-methods approach, integrating quantitative VAS coverage and cost data, a cost-effectiveness analysis, and qualitative insights from stakeholders to evaluate the efficiency of VAS delivery approaches during the second semester of 2023. The study took place between January and May 2024, with data collection between January and March 2024 involving 20 local structures and nine national and international organizations.

The National Ethics Committee for Health and Life Sciences (CNESVS), Côte d’Ivoire, approved the study (Reference No. 00001_24/MSHPCMU/CNESVS-km). Following the study’s established ethical protocols, informed consent was obtained in writing before each interview.

### Study setting

The study was conducted in two health districts in northern Côte d’Ivoire: Ferkessédougou, located in the Tchologo region, and Niakaramadougou, in the Hambol region. These districts were strategically selected based on their logistical feasibility and representation of diverse geographical and infrastructural contexts relevant to routine VAS implementation.

To be included in the study, each district must implement routine VAS during the study period, have sufficient population size to meet the sampling requirements of the post-event coverage survey (PECS), with at least 123 enumeration areas, and have a historical VAS coverage of 80% or higher during campaign-based delivery strategies.

During the second semester of 2023 (July 1 – December 31), the two districts implemented different combinations of routine-fixed, advanced, and catch-up strategies:

- Ferkessédougou:
  - A catch-up campaign was conducted in July 2023.
  - Routine-fixed distribution from August to September 2023.
  - Advanced strategy distribution from October to December 2023.
- Niakaramadougou:
  - A catch-up campaign was conducted in July 2023.
  - Routine-fixed distribution from August to November 2023.
  - Another catch-up campaign occurred in December 2023.

The July 2023 catch-up campaign was originally scheduled for semester 1 (June 2023) but was delayed due to unforeseen logistical challenges, which impacted the schedule for routine-fixed VAS distribution in both districts.

The districts exhibited distinct geographical and infrastructural characteristics that affected VAS delivery. Ferkessédougou and Niakaramadougou are different types of settings, in that Ferkessédougou has a more urbanized infrastructure (roads, transportation networks), which enables better access to healthcare. However, it faces challenges due to overcrowding and other socio-economic barriers. Conversely, Niakaramadougou, a predominantly rural district, experiences logistical challenges such as poor road conditions and limited health infrastructure, especially during the rainy season from April to October.

Although the national rollout of advanced strategies is based on multiple criteria, including VAS performance, geographic accessibility, CHW network readiness, operational capacity, and available partner financing, the difference observed between the two study districts during this semester was primarily due to operational funding. Ferkessédougou benefited from partner-supported financing that allowed advanced outreach, whereas Niakaramadougou did not receive comparable support during this period and therefore implemented only routine-fixed and catch-up activities.

### Study setting and population

The health districts of Ferkessédougou and Niakaramadougou have a combined estimated population of 50470 children aged 6–59 months (49.4% in Ferkessédougou and 50.6% in Niakaramadougou). The population distribution varies between rural and urban settings, with approximately 60% residing in rural areas and 40% in urban areas.

The study population consisted of two key groups: caregivers of children aged 6–59 months and health actors at various levels of the healthcare system. A PECS was employed to assess VAS coverage. A two-stage cluster sampling methodology, stratified by rural and urban areas, was employed in accordance with the World Health Organization’s (WHO) 2018 immunization coverage survey guidelines [9]. For cost data, a non-probabilistic purposive sampling approach was used to collect information from 91 stakeholders across 16 health facilities, two regional and two departmental offices, and nine partner organizations that contributed data on the operational and financial aspects of the VAS program in both districts. This sample represented approximately 94% (29 of 31) of the organizations working to combat micronutrient deficiencies, malnutrition, and related health services in the two districts.

### Data collection

Data collection for this study focused on two primary types of data: VAS program costs and VAS coverage. Cost data were collected in two phases: regional/district (January 24-February 8, 2024) and national (February 14-March 19, 2024). Semi-structured face-to-face interviews were the main data collection method; when respondents were unavailable for in-person meetings, they completed the questionnaires via email.

The PECS was conducted from January 25 to February 8, 2024. Research assistants used tablets equipped with mobile data collection tools (ODK and ONA) for real-time and secure data entry. Supervisors re-surveyed 10% of households to ensure data accuracy, with discrepancies addressed through follow-up surveys. Table 1 shows the number of caregivers surveyed with response rates.

**Table 1:** Number of caregivers surveyed with response rates.

	Ferkessédougou	Niakaramadougou
Number of mothers/caregivers interviewed	1,384	1,397
Number of cases of mother/caregiver refusals	41	7
Response rate	97.1%	99.5%

### Costing approach

To estimate the full economic and financial costs of the VAS delivery strategies, we used a combined top-down and bottom-up costing approach, integrating a full cost method with an ingredients-based method.

### Integration of costing methods

The ingredients method was used to estimate direct implementation costs by quantifying specific inputs (e.g., CHW time, transport, supplies) and multiplying them by their unit costs. Unit costs were derived from financial records, standardized government salary scales, and partner-provided market rates. At the same time, the full cost method classified all expenditures into direct or indirect categories and ensured that system-wide costs, such as administrative overhead, planning, or infrastructure, were incorporated.

The two approaches were integrated by mapping all cost data to six key VAS activities (planning, procurement, training, social mobilization, distribution, supervision/monitoring) and four cost categories (personnel, transport, supplies, administration). Costs were further disaggregated by:

- Level of implementation (national, regional, district, health facility)
- Strategy (routine-fixed, advanced, catch-up)
- Geographic setting (urban vs. rural)
- Cost type (direct or indirect)

This framework allowed each cost line to be classified and, where necessary, disaggregated using standardized parameters.

### Apportionment of indirect costs

Indirect costs, such as administrative salaries and shared office costs, were allocated to the VAS program based on structured time-use interviews with 91 stakeholders across levels. Respondents estimated the proportion of time dedicated to VAS activities during the second semester of 2023. For each respondent, a daily wage was calculated by dividing their gross monthly salary (including benefits) by 22 working days. Their total VAS-attributed cost was calculated by multiplying the time dedicated to each VAS component by their daily wage, and summing across strategies.

For national-level actors supporting all 73 districts, district-level allocation was performed using the relative weight of VAS in each district (e.g., 1.0% for Ferkessédougou and 1.1% for Niakaramadougou). These weights were based on the share of the district’s target population and historical program size. Equipment costs were amortized based on standard linear depreciation rates (e.g., 20% for vehicles, 33% for IT equipment).

### Cost disaggregation process

To ensure precision in assigning costs by delivery modality, all shared and indirect costs were progressively disaggregated by strategy, location, and VAS component using validated weighting parameters. For example, fuel cost incurred centrally was disaggregated proportionally by rural-urban child population shares (e.g., 59.0% rural in Ferkessédougou), then further by strategy use (e.g., 68.4% for the advanced strategy). This process was applied to all cost records, ensuring complete attribution of each cost item.

When full disaggregation variables were unavailable, costs were split using imputation coefficients derived from PECS or DHIS2 data.

### Personnel and other cost treatment

Personnel costs were treated separately through a multi-step process:

1. Daily wages were calculated for each actor.
2. Full-time equivalents (FTEs) were estimated based on reported time on each VAS strategy and component.
3. These were multiplied to generate individual personnel costs, summed by activity, and then aggregated by district.

In this study, facility-based health staff primarily delivered VAS through the routine-fixed strategy within health centers, while CHWs supported outreach activities through the advanced and catch-up strategies. However, in certain remote sub-districts, CHWs were temporarily mobilized to assist fixed-site sessions due to workforce shortages. This represents a context-specific adaptation, rather than a deviation from standard integrated service delivery norms, and is noted to avoid misinterpretation when extrapolating results to national scale.

When salary data were missing, median wages for comparable positions were used.

Other costs (supplies, transport, administration) were calculated using the same matrix-based approach and fully disaggregated using consistent parameters. Notably, the cost of vitamin A capsules and delivery materials was classified under procurement, not distribution, to reflect centralized sourcing and ensure accurate categorization.

All disaggregated cost lines were compiled into a unified Excel-based database containing the following fields: district, VAS strategy, reporting organization, level of intervention, residence type (urban/rural), VAS component, cost category, description, total cost, funding source, and share financed.

### Cost-effectiveness analysis

The cost-effectiveness analysis evaluated two main outcomes: the total cost per child supplemented with vitamin A, and the cost per disability-adjusted life year (DALY) averted through the three routine VAS strategies. The total cost per child supplemented was calculated by dividing the total program costs by the number of children who received VAS (as determined by the PECS). DALYs represent the health burden that an intervention seeks to prevent [10]. In this context, VAS reduces VAD and thereby lowers the incidence and severity of VAD-related illnesses such as diarrhea and measles among young children [11].

The DALY calculations followed parameters from the 2023 Global Burden of Disease (GBD) study and incorporated updated disability weights for both diarrhea and measles [12]. DALYs were computed as the sum of the years lived with disability (YLD) due to morbidity and the years of life lost (YLL) due to premature mortality.

YLD was calculated using the formula:


YLD=Number of cases (diarrhea and measles)×Duration until remission or death×Disability weight

YLL was calculated as:


YLL=Number of deaths from diarrhea and measles×Life expectancy at ages less than 5 years

A standard life expectancy of 66.1 years for children aged less than 5 years was taken from the Côte d’Ivoire life table [13]. The DALYs averted by the VAS program were estimated by comparing the baseline DALYs lost due to diarrhea and measles in the absence of VAS with the DALYs expected under the VAS intervention. Table 2 summarizes the key assumptions and parameter sources used in the DALY calculations, including life expectancy, disability weights, and mortality reduction factors. All estimates were derived from the Global Burden of Disease (GBD) 2023 database [13], and the most recent meta-analyses on VAS and child mortality [4].

**Table 2:** Parameters for DALY calculation and sources.

Variables	Value	Source
Life expectancy (Children <5 years old)	66.1	Global Burden of Disease (GBD) 2023, Institute for Health Metrics and Evaluation.
disability weights	Measles: 0.13 Diarrhea: 0.24	
Mortality rate	Measles: 78.78/100,000 Diarrhea: 24.43/100,000	
VAS all-cause mortality reductions.	0.24	Imdad et al. 2017 meta-analysis [4]

The cost per DALY averted was assessed against a threshold of USD 1,265 (equivalent to 0.5 times Côte d’Ivoire’s per capita GDP in 2023) [14,15]. This value served as a proxy benchmark to determine whether the three different routine VAS delivery strategies could be considered cost-effective. A one-way sensitivity analysis was conducted by varying key parameters (personnel costs, the relative mortality reduction attributable to VAS, and VAS coverage) using ±25% adjustments.

## Results

### Total program costs

The total cost of the VAS program was analyzed by health district, urban/rural setting, and delivery strategy to provide a detailed understanding of the program’s financial structure. For the second half of 2023, the total VAS program cost amounted to 25,519,631 FCFA. Of this, 47.2% of costs were incurred in Ferkessédougou (12,053,476 FCFA), while 52.8% were from Niakaramadougou (13,466,155 FCFA). The VAS delivery strategies employed in rural areas incurred higher expenditures compared to urban areas (60% and 40%, respectively).

Disaggregation by delivery strategy showed that the catch-up strategy in Niakaramadougou in December 2023 was the most expensive approach, costing 5,580,254 FCFA, with 59.6% of expenditures occurring in rural areas. In contrast, the routine-fixed distribution across both districts had the lowest costs, totaling 4,243,988 FCFA, with costs higher in Niakaramadougou (2,999,460 FCFA) than in Ferkessédougou (1,244,528 FCFA). In Ferkessédougou, the advanced strategy resulted in a total cost of 5,607,278 FCFA.

Table 3 provides an overview of program costs across districts and delivery approaches, highlighting the rural-urban cost split.

**Table 3:** Overall VAS program costs per district and distribution strategy (FCFA).

Delivery Strategy	Ferkéssedougou		Niakaramadougou		Total	
	Rural	Urban	Rural	Urban	Rural	Urban
Catch-up (Jul 2023)	3,071,066	2,130,604	2,910,305	1,976,135	5,981,371	4,106,740
Fixed	721,810	522,718	1,784,008	1,215,451	2,505,818	1,738,169
Advanced	3,323,496	2,283,781	–	–	3,323,496	2,283,781
Catch-up (Dec 2023)	–	–	3,324,329	2,255,924	3,324,329	2,255,924
**Total**	**7,116,372**	**4,937,103**	**8,018,642**	**5,447,512**	**15,135,014**	**10,384,616**

### Key cost drivers

In Ferkéssedougou, distribution costs accounted for the highest expenditure in catch-up and advanced distribution approaches. However, supervision and monitoring costs were the most significant for the fixed approach, exceeding distribution expenses by nearly 25%. This cost pattern was consistent across both urban and rural settings. In Niakaramadougou, distribution costs were the highest for the July 2023 catch-up approach. While distribution remained a significant expense for the December 2023 catch-up event and the fixed approach, supervision and monitoring costs were the highest among all activities. This trend was observed in both urban and rural settings. Overall, rural activity costs in both districts were consistently higher than in urban areas. These cost distributions are detailed in Appendix 1, which presents the overall VAS program costs by activity type, district, urban/rural setting, and distribution approach.

Personnel costs consistently represented the largest and most significant expenditure for all delivery modalities, accounting for over 70% of total VAS program costs for semester two, 2023 (July 1 – December 31, 2023). These included salaries and benefits for health workers who distribute VAS in the health centers as part of the routine-fixed strategy and CHWs who distribute VAS in the routine advanced and catch-up strategy. Transportation costs were also significant, particularly in rural areas where poor infrastructure often increases travel times and requires specialized vehicles to access more dispersed communities and households.

### VAS coverage

Differences in implementation schedules and delivery approaches resulted in the cohorts of children eligible for VAS varying from one month to the next. These shifts in target populations resulted in corresponding variations in the denominator used to assess coverage across different strategies. VAS coverage varied significantly across districts and delivery approaches (Table 4).

**Table 4:** Number of children supplemented and coverage by district and delivery approach.

Indicator	Ferkessédougou					Niakaramadougou				
Number	Rural	Urban	Total	Target	Coverage (%)	Rural	Urban	Total	Target	Coverage (%)
**Catch-up (Jul 2023)**	1,906	1,422	3,328	22,945	14.5	2,003	1,282	3,285	22,645	14.5
**Fixed**	1,211	723	1,934	21,419	8.7	2,500	2,085	4,585	20,421	20.4
**Advanced**	7,249	4,154	11,403	20,845	51.2	–	–	–	–	0.0
**Catch-up (Dec 2023)**	–	–	–	–	0.0	5,483	2,826	8,309	19,193	43.3
**Total**	10,365	6,299	16,665	24,908	66.9	9,986	6,193	16,180	25,562	63.3

In Ferkessédougou, the routine-fixed distribution achieved 8.7% coverage, while Niakaramadougou attained a higher coverage of 20.4%. Among all delivery strategies employed in both districts, the advanced strategy in Ferkessédougou attained the highest coverage (51.2%), followed by the catch-up strategy implemented in December 2023, which achieved 43.3% coverage.

### Cost per child supplemented

Similar to VAS coverage, the cost per child supplemented varied across districts, delivery strategies, and geographic settings (Table 5).

**Table 5:** Cost per child supplemented by district, urban/rural setting, and VAS delivery approach (FCFA).

Routine VAS Distribution Strategy	Ferkéssedougou		Niakaramadougou		Total	
	Rural	Urban	Rural	Urban	Rural	Urban
Catch-up (Jul 2023)	1,611	1,497	1,452	1,541	1,530	1,518
Fixed	596	723	713	582	675	619
Advanced	458	549	–	–	458	549
Catch-up (Dec 2023)	–	–	606	798	606	798
**Total**	**686**	**783**	**803**	**879**	**743**	**831**

In Ferkessédougou, the routine advanced strategy was the most cost-effective, achieving the lowest cost per child supplemented across all strategies, at 458 FCFA in rural areas and 549 FCFA in urban areas. The routine-fixed strategy was followed, with costs of 596 FCFA in rural areas and 723 FCFA in urban areas. In contrast, the routine catch-up strategy implemented in July 2023 was the most expensive, with the cost per child supplemented reaching 1,611 FCFA in rural areas and 1,497 FCFA in urban areas.

In Niakaramadougou, where the advanced strategy was not implemented, the routine-fixed distribution strategy had a cost per child of 713 FCFA in rural areas and 582 FCFA in urban areas. Among the two routine catch-up events conducted in July and December 2023, the December catch-up event proved more cost-effective in rural settings, with a cost of 606 FCFA per child, lower than the routine-fixed strategy in rural areas. However, in urban areas, the routine-fixed strategy remained more cost-effective (582 FCFA) than both catch-up events, with the December catch-up costing 798 FCFA per child.

### Allocation of VAS program costs across the health system

An analysis of VAS program costs across different health system levels revealed that most expenditures (68%) occurred at the community (aire de santé) level (Table 6).

**Table 6:** VAS program cost by activity type, VAS cost component, and the location where costs were incurred (FCFA).

Cost Categories per Activity	Aire de santé	District	National	Regional
**Administrative costs**	**–**	**105,498**	**55,596**	**–**
Procurement	–	14,698	6,986	–
Training	–	5,977	4,117	–
Social Mobilization	–	17,715	3,894	–
Planning	–	12,349	10,264	–
Supervision & Monitoring	–	54,757	30,334	–
**Supplies**	**2,365,780**	**675,950**	**172,545**	**1,111**
Procurement	2,176,312	12,671	20,916	–
Distribution	79,613	–	–	–
Training	13,878	5,153	11,241	–
Social Mobilization	62,417	125,272	57,741	–
Planning	4,542	10,646	28,721	–
Supervision & Monitoring	29,015	522,206	53,924	1,111
**Personnel**	**13,372,805**	**3,732,400**	**1,773,551**	**221,954**
Procurement	654,074	346,665	184,236	–
Distribution	6,359,171	–	–	–
Training	665,334	199,081	169,430	–
Social Mobilization	4,106,078	632,260	129,089	–
Planning	230,611	530,289	477,701	29,895
Supervision & Monitoring	1,357,534	2,024,103	813,094	192,059
**Transport**	**2,226,000**	**366,884**	**445,662**	**3,888**
Procurement	216,000	12,546	8,566	–
Distribution	2,010,000	–	–	–
Training	–	217,472	4,731	–
Social Mobilization	–	7,328	4,783	–
Planning	–	5,108	11,998	–
Supervision & Monitoring	–	124,428	415,582	3,888
**Total Cost**	**17,964,585**	**4,880,734**	**2,447,356**	**226,954**

These community-level costs were driven primarily by personnel expenses (13,372,805 FCFA) and distribution activities (6,359,171 FCFA). Supplies and transportation were also substantial contributors, with supply costs totaling 2,365,780 FCFA and transportation costs amounting to 2,226,000 FCFA.

District-level expenditures accounted for 18% of the total program costs, with significant allocations to supervision and monitoring activities (22,725,495 FCFA) and planning (558,395 FCFA). Social mobilization also represented a considerable expense at the district level, amounting to 782,576 FCFA. National-level costs represented 13% of the total expenditure, primarily for personnel (1,773,551 FCFA), supervision and monitoring (1,312,935 FCFA), and planning activities (528,686 FCFA).

Regional level costs were minimal, accounting for 1% of the total budget. The most significant expenditures were supervision and monitoring (197,059 FCFA) and personnel costs (29,895 FCFA).

In Ferkessédougou, the routine advanced strategy has the lowest cost per DALY averted (30,093 FCFA). The cost per DALY averted suggests that the routine advanced strategy is highly cost-effective compared to other routine strategies in the two study regions (Table 7). In Niakaramadougou, the second routine catch-up (Dec 2023) has the lowest cost per DALY averted. In the two regions, the routine catch-up (July 2023) has the highest cost per DALY averted, indicating a lesser impact and return on the supplementation process.

**Table 7:** Cost-effectiveness (Cost per DALY averted) by strategy.

	Ferkessédougou		Niakaramadougou		Overall	
Method	DALYs Averted	Cost per DALY Averted (FCFA)	DALYs Averted	Cost per DALY Averted (FCFA)	DALYs Averted	Cost per DALY Averted (FCFA)
**Catch-up (Jul 2023)**	58	89,550	57	85,238	574	44,488
**Fixed**	33	38,253	73	41,240		
**Advanced**	186	30,093	–	–		
**Catch-up (Dec 2023)**	–	–	145	38,460		
**Total**	291	41,431	283	47,668		

The analysis shows that the cost per DALY averted (44,488 FCFA) is well below Côte d’Ivoire’s cost-effectiveness threshold.

### Sensitivity analysis

**Fig 1:**
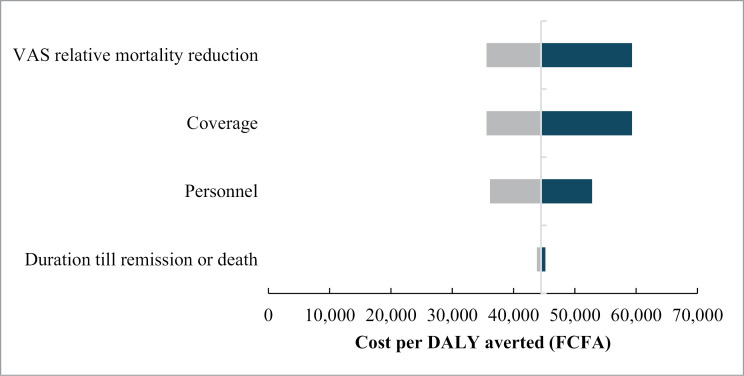
Tornado diagram of one-way sensitivity analysis showing the impact of different variables on the cost per DALY averted.

As shown in Fig 1, the one-way sensitivity analysis revealed that the VAS relative mortality reduction and VAS coverage have a relatively high impact on the cost per DALY averted. The higher the effectiveness of VAS and the coverage, the lower the cost per DALY averted. The parameters on the left are ranked in order of influence on the cost per DALY.

## Discussion

Consistent with findings from Laillou et al. (2024), Janmohamed et al. (2017, 2024), and Horton et al. (2018), our study found significant variation in VAS coverage across districts, delivery strategies, and geographic settings [16–19]. The routine-fixed delivery strategy had the lowest coverage in both study districts, while more resource-intensive strategies, particularly the advanced strategy in Ferkessédougou and the December catch-up in Niakaramadougou, demonstrated substantially higher reach.

The marked differences between districts and catch-up rounds reflect operational realities rather than inherent differences in strategy effectiveness. The absence of the advanced strategy in Niakaramadougou during the study period stemmed from limited partner-supported operational financing, despite the district’s eligibility under national policy. In contrast, Ferkessédougou benefited from external funding that enabled consistent implementation of advanced outreach activities. District-level financing and resource availability continue to shape the pace and feasibility of transitioning from campaign-based to routine VAS delivery.

Similarly, the large discrepancy in catch-up coverage between rounds in Niakaramadougou was driven by context-specific implementation factors. The July 2023 catch-up faced constraints including the lack of central-level supervision, reduced CHW participation due to unpaid incentives, and limited engagement from community leaders. In contrast, the December round benefitted from strengthened supervision, improved coordination, and a structured operational roadmap, resulting in markedly higher coverage. These differences highlight the importance of consistent supervision, which aligns with previous evidence from Ezezika et al. (2024) [20] and Berihun et al. (2023) [21] demonstrating the role of operational quality in shaping intervention uptake, timely remuneration, and community engagement in achieving effective routine VAS delivery.

Geographic disparities were evident across delivery strategies. Rural coverage consistently exceeded urban coverage, for example, 55.0% versus 45.7% in Ferkessédougou and 48.0% versus 36.4% in Niakaramadougou – despite greater geographic barriers to service delivery. Similar rural–urban patterns have been reported in studies by Janmohamed et al. and Ezezika [17,19,20]. In our study, this pattern appears driven less by access constraints and more by differences in social mobilization investments, which were substantially lower in urban settings. While urban caregivers may have better physical access to health facilities, insufficient mobilization may reduce awareness and uptake. These findings reinforce the need for tailored communication approaches across geographic settings.

Indirect costs, while proportionately modest, remained an integral component of overall program expenditure. These costs reflected shared use of health system infrastructure, administrative support, and utilities across programs. Integrating routine VAS within existing primary health care structures allowed these indirect costs to be distributed more efficiently, supporting long‑term sustainability and aligning with national goals for health systems integration.

Personnel costs dominated expenditures across all strategies, reflecting the labor-intensive nature of VAS delivery, consistent with findings from Neidecker-Gonzales et al. (2007) [22], Kagin et al. (2015) [23], and Saitowitz et al. (2001) [24]. Routine-fixed strategies were generally the least costly per child supplemented but demonstrated limited reach. In contrast, catch-up and advanced strategies extended coverage through proactive community engagement, though at higher cost. The advanced strategy in Ferkessédougou achieved the best balance of cost and reach, suggesting it may offer a sustainable middle-ground model when adequate financing and CHW capacity are available.

The cost per DALY averted across all strategies remained well below the national cost-effectiveness threshold, indicating that routine VAS delivery, regardless of modality, represents a highly cost-effective child health intervention. Sensitivity analyses revealed that VAS effectiveness and coverage are the primary drivers of cost per DALY averted, suggesting that expanding and sustaining high coverage levels would yield the greatest gains in cost-effectiveness.

### Policy implications

Effective VAS delivery in Côte d’Ivoire requires aligning strategy selection with district-level operational capacity, financing availability, and geographic accessibility. Routine-fixed strategies may remain a practical default for districts with limited resources, while advanced outreach offers clear advantages in improving coverage in rural and underserved populations. Catch-up strategies still play a meaningful role in closing gaps, but should be targeted and used judiciously given their higher cost.

Strengthening supervision systems, ensuring timely CHW incentive payments, and increasing social mobilization in urban settings are critical operational priorities for improving routine VAS effectiveness. A phased scale-up of advanced strategies, prioritizing districts with appropriate readiness and financing, aligns with national health system strengthening goals and can support sustainable progress.

### Feasibility, scalability, and sustainability

National scale-up of routine advanced outreach will depend on the availability of trained CHWs, reliable transport and supply chains, and stable financing for operational costs. Districts with dispersed rural populations should be prioritized for advanced outreach, while urban districts may benefit from strengthening routine-fixed strategies through enhanced mobilization. These adjustments align with WHO principles for integrated, equity-oriented service delivery.

### Strengths and limitations

While our study provides valuable insights into routine VAS delivery strategies’ operational and financial efficiency in two distinct health districts, several limitations should be noted. First, the study was limited to two districts, Ferkessédougou and Niakaramadougou, which may affect the generalizability of our findings. Nevertheless, the two districts’ diverse geographic and operational contexts are representative of the range of implementation settings in Côte d’Ivoire and support their contribution to national policy discussions.

Another limitation was the reliance on stakeholder interviews to collect data on time and resource allocation for the VAS program. This data collection method may have introduced recall bias. We aimed to mitigate this limitation by triangulating data from administrative records, program guidelines, and financial documents.

The study did not account for indirect costs borne by caregivers, such as time spent and out-of-pocket expenses incurred when accessing services. These expenses represent a significant societal burden and barrier to accessing VAS and should be included in future cost-effectiveness research.

Finally, the overlapping implementation of routine-fixed and catch-up strategies in July 2023 poses challenges for distinguishing the costs associated with the specific delivery strategies. Despite this, data from other months were robust, ensuring that the studies’ conclusions were reliable.

## Conclusions

This study provides new, context-specific evidence on the costs and operational performance of routine VAS delivery strategies in two health districts in Côte d’Ivoire. By integrating coverage, cost, and implementation data, the analysis offers practical insights for districts navigating the ongoing transition from campaign-based to routine delivery. Although routine-fixed distribution remained the least costly approach, its comparatively low reach demonstrates the importance of complementary strategies. In particular, the advanced strategy demonstrated stronger coverage and favorable cost-effectiveness in rural settings where operational support and CHW capacity were sufficient.

Implementation conditions influenced performance across districts, such as the availability of operational financing, the intensity of supervision, and CHW engagement, shape the feasibility and performance of routine VAS delivery. These operational drivers were central to differences observed between districts and across catch-up rounds, emphasizing the need to account for local capacity when selecting and planning delivery strategies.

Overall, the results suggest opportunities to enhance routine VAS delivery through improved supervision systems, predictable operational financing, and more tailored social mobilization efforts, particularly in urban settings where uptake was lower. Expanding similar analyses to additional districts will be important to confirm these patterns and further inform national planning for a sustainable and equitable routine VAS program.

## Supporting information

S1 Table Overall VAS program costs by activity type, health district, urban/rural settings and distribution approach (FCFA). (DOCX)
