# Supplementary material for: Optimizing vitamin A supplementation: A comparative cost-effectiveness analysis of routine distribution strategies in northern Côte d’Ivoire
Source: PLoS One. 2025 Dec 15;20(12):e0338784. doi: 10.1371/journal.pone.0338784 (PMC12704894; doi:10.1371/journal.pone.0338784)
Supplement: S1 Table — (DOCX) [file pone.0338784.s001.docx]

**Supporting Information, Table 1 - Overall VAS program costs by activity type, health district, urban/rural settings and distribution approach (FCFA)**

| **Cost Categories and Components** | **Ferkéssedougou** | | | | | | **Niakaramadougou** | | | | | |
| --- | --- | --- | --- | --- | --- | --- | --- | --- | --- | --- | --- | --- |
|  | **Rural** | | | **Urban** | | | **Rural** | | | **Urban** | | |
|  | **Catch-up**  **(Jul 2023)** | **Fixed** | **Advanced** | **Catch-up**  **(Jul 2023)** | **Fixed** | **Advanced** | **Catch-up**  **(Jul 2023)** | **Catch-up**  **(Dec 2023)** | **Fixed** | **Catch-up**  **(Jul 2023)** | **Catchup**  **(Dec 2023)** | **Fixed** |
| **Procurement** | **344,008** | **113,377** | **521,083** | **238,661** | **78,657** | **361,510** | **372,739** | **569,151** | **248,039** | **252,905** | **385,617** | **167,921** |
| Administrative | 1,276 | 726 | 4,281 | 885 | 503 | 2,970 | 1,326 | 3,357 | 1,902 | 897 | 2,271 | 1,286 |
| Supplies | 154,543 | 59,406 | 367,301 | 107,217 | 41,214 | 254,821 | 201,926 | 360,695 | 168,676 | 136,428 | 243,702 | 113,966 |
| Personnel | 171,835 | 43,767 | 93,610 | 119,213 | 30,364 | 64,943 | 157,535 | 174,836 | 60,778 | 107,504 | 119,194 | 41,393 |
| Transport | 16,352 | 9,478 | 55,89 | 11,344 | 6,575 | 38,774 | 11,951 | 30,261 | 16,683 | 8,076 | 20,450 | 11,274 |
| **Distribution** | **964,507** | **175,623** | **1,808,489** | **669,143** | **121,841** | **1,254,670** | **911,820** | **817,266** | **330,133** | **616,884** | **553,002** | **225,403** |
| Supplies | 3,631 | 3,352 | 19,765 | 2,519 | 2,325 | 13,712 | 3,725 | 9,433 | 7,315 | 2,517 | 6,373 | 4,942 |
| Personnel | 825,084 | 172,271 | 938,548 | 572,416 | 119,516 | 651,133 | 806,639 | 706,376 | 322,817 | 545,822 | 478,085 | 220,460 |
| Transport | 135,792 | - | 850,176 | 94,208 | - | 589,824 | 101,456 | 101,456 | - | 68,544 | 68,544 | - |
| **Training** | **202,989** | **56,662** | **119,749** | **140,827** | **39,310** | **83,078** | **164,904** | **73,258** | **151,070** | **111,723** | **49,813** | **103,032** |
| Administrative | 313 | 506 | 2,989 | 217 | 351 | 2,073 | 330 | 837 | 1,004 | 223 | 566 | 679 |
| Supplies | 1,248 | 1,245 | 7,345 | 866 | 864 | 5,096 | 1,307 | 3,310 | 3,496 | 885 | 2,242 | 2,365 |
| Personnel | 128,798 | 54,598 | 107,579 | 89,356 | 37,878 | 74,635 | 107,738 | 68,291 | 145,893 | 73,098 | 46,448 | 99,529 |
| Transport | 72,628 | 311 | 1,835 | 50,387 | 216 | 1,273 | 55,527 | 819 | 676 | 37,515 | 555 | 457 |
| **Social mobilization** | **849,454** | **88,386** | **165,070** | **589,323** | **61,319** | **114,520** | **780,044** | **797,072** | **372,764** | **531,344** | **542,857** | **254,424** |
| Administrative | 1,777 | 459 | 2,710 | 1,232 | 318 | 1,880 | 1,832 | 4,639 | 1,422 | 1,238 | 3,136 | 961 |
| Supplies | 16,607 | 2,460 | 14,505 | 11,521 | 1,706 | 10,063 | 47,151 | 56,841 | 8,534 | 31,858 | 38,411 | 5,770 |
| Personnel | 830,150 | 85,170 | 146,105 | 575,931 | 59,088 | 101,363 | 730,107 | 733,176 | 361,947 | 497,601 | 499,676 | 247,108 |
| Transport | 918 | 296 | 1,748 | 637 | 205 | 1,213 | 953 | 2,414 | 861 | 645 | 1,634 | 583 |
| **Planning** | **114,394** | **68,748** | **188,893** | **79,362** | **47,695** | **131,047** | **107,982** | **159,475** | **162,242** | **73,509** | **108,316** | **110,462** |
| Administrative | 1,098 | 879 | 5,186 | 762 | 610 | 3,598 | 1,151 | 2,914 | 2,184 | 779 | 1,972 | 1,477 |
| Supplies | 2,345 | 1,597 | 9,416 | 1,627 | 1,107 | 6,532 | 2,473 | 6,262 | 3,947 | 1,677 | 4,247 | 2,676 |
| Personnel | 110,046 | 65,648 | 170,611 | 76,346 | 45,544 | 118,364 | 103,404 | 147,882 | 154,540 | 70,406 | 100,458 | 105,245 |
| Transport | 903 | 623 | 3,679 | 626 | 432 | 2,552 | 954 | 2,416 | 1,569 | 647 | 1,638 | 1,064 |
| **Supervision & Monitoring** | **595,713** | **219,011** | **520,211** | **413,286** | **173,892** | **338,955** | **572,813** | **908,106** | **519,757** | **389,768** | **616,316** | **354,208** |
| Administrative | 6,564 | 2,455 | 14,481 | 4,554 | 1,703 | 10,046 | 6,831 | 14,168 | 6,021 | 4,616 | 9,576 | 4,070 |
| Supplies | 73,051 | 4,446 | 86,067 | 50,680 | 20,029 | 42,765 | 82,019 | 79,657 | 34,773 | 55,421 | 53,838 | 23,505 |
| Personnel | 480,171 | 210,645 | 410,387 | 333,126 | 150,695 | 280,157 | 446,884 | 578,695 | 474,205 | 304,677 | 393,731 | 323,414 |
| Transport | 35,925 | 1,463 | 9,275 | 24,924 | 1,464 | 5,986 | 37,077 | 235,585 | 4,756 | 25,053 | 159,170 | 3,218 |
